# Comprehensive analysis of tumor immune microenvironment and prognosis of m6A-related lncRNAs in gastric cancer

**DOI:** 10.1186/s12885-022-09377-8

**Published:** 2022-03-24

**Authors:** Yi Wang, Gui-Qi Zhu, Di Tian, Chang-Wu Zhou, Na Li, Ying Feng, Meng-Su Zeng

**Affiliations:** 1 Department of Radiology, Zhongshan Hospital, Fudan University, Shanghai Institute of Medical Imaging, Xuhui District, Shanghai, 200032 China; 2 Department of Liver Surgery and Transplantation, Liver Cancer Institute, Zhongshan Hospital, Fudan University, Shanghai, 200032 China; 3 Department of Gastrointestinal Surgery, Affiliated Hospital of Nantong University, 20 Xisi Street, Nantong, 226000 Jiangsu China

**Keywords:** N6-methyladenosine, Long non-coding RNAs, Tumor immune microenvironment, Prognosis, Immune checkpoint inhibitors therapy, Gastric cancer

## Abstract

**Background:**

N6-methyladenosine (m6A) modification and long non-coding RNAs (lncRNAs) play pivotal roles in gastric cancer (GC) progression. The emergence of immunotherapy in GC has created a paradigm shift in the approaches of treatment, whereas there is significant heterogeneity with regard to degree of treatment responses, which results from the variability of tumor immune microenvironment (TIME). How the interplay between m6A and lncRNAs enrolling in the shaping of TIME remains unclear.

**Methods:**

The RNA sequencing and clinical data of GC patients were collected from TCGA database. Pearson correlation test and univariate Cox analysis were used to screen out m6A-related lncRNAs. Consensus clustering method was implemented to classify GC patients into two clusters. Survival analysis, the infiltration level of immune cells, Gene set enrichment analysis (GSEA) and the mutation profiles were analyzed and compared between two clusters. A competing endogenous RNA (ceRNA) network and Kyoto Encyclopedia of Genes and Genomes (KEGG) pathway analysis were applied for the identification of pathways in which m6A-related lncRNAs enriched. Then least absolute shrinkage and selection operator (LASSO) COX regression was implemented to select pivotal lncRNAs, and risk model was constructed accordingly. The prognosis value of the risk model was explored. In addition, the response to immune checkpoint inhibitors (ICIs) therapy were compared between different risk groups. Finally, we performed qRT-PCR to detect expression patterns of the selected lncRNAs in the 35 tumor tissues and their paired adjacent normal tissues, and validated the prognostic value of risk model in our cohort (*N* = 35).

**Results:**

The expression profiles of 15 lncRNAs were included to cluster patients into 2 subtypes. Cluster1 with worse prognosis harbored higher immune score, stromal score, ESTIMATE score and lower mutation rates of the genes. Different immune cell infiltration patterns were also displayed between the two clusters. GSEA showed that cluster1 preferentially enriched in tumor hallmarks and tumor-related biological pathways. KEGG pathway analysis found that the target mRNAs which m6A-related lncRNAs regulated by sponging miRNAs mainly enriched in vascular smooth muscle contraction, cAMP signaling pathway and cGMP-PKG signaling pathway. Next, eight lncRNAs were selected by LASSO regression algorithm to construct risk model. Patients in the high-risk group had poor prognoses, which were consistent in our cohort. As for predicting responses to ICIs therapy, patients from high-risk group were found to have lower tumor mutation burden (TMB) scores and account for large proportion in the Microsatellite Instability-Low (MSI-L) subtype. Moreover, patients had distinct immunophenoscores in different risk groups.

**Conclusion:**

Our study revealed that the interplay between m6A modification and lncRNAs might have critical role in predicting GC prognosis, sculpting TIME landscape and predicting the responses to ICIs therapy.

**Supplementary Information:**

The online version contains supplementary material available at 10.1186/s12885-022-09377-8.

## Introduction

Gastric cancer (GC) is the fifth most lethal tumor and estimated to be the third most common cause of cancer-related death [1, 2]. Accumulating researches have suggested that epigenomic alterations acted as a crucial role through activation of oncogenes or tumor suppressor genes in the gastric carcinogenesis [3, 4]. Presently, N6-methyladenosine (m6A) is the most common RNA modifications, which was found not only in mRNAs, but also in ncRNAs, such as microRNAs (miRNAs), long non-coding RNAs (lncRNAs) and circular RNAs (circRNAs) through modulating the splicing, stability and translation of ncRNAs [5]. Intriguingly, non-coding RNAs were also demonstrated to have regulatory role for the expression of m6A regulatory proteins. Therefore, the interaction between m6A and noncoding RNAs exerts a synergistic effect on carcinogenesis and provides novel cancer treatment strategies [6–8]. To note, according to the previous researches, the interplay models between m6A modification and lncRNAs in tumor were diverse and complex. For instance, miR503HG promoted the degradation of HNRNPA2B to inhibit HCC migration via reducing the stability of p52 and p65 mRNA [9]. GATA3-AS acted as a guide lncRNA promoting the m6A modification of GATA3 pre-mRNA by KIAA 1429, thereby down-regulating the expression of GATA3, which contributed to the growth and the metastasis of HCC [10]. Recently, with the increased understanding of the diversity of tumor microenvironment (TME), cross-talk between tumor cells and surrounding cells plays a crucial role in the tumor progression [11]. Meanwhile, m6A modification was reported to be critically associated with tumor immune microenvironment (TIME) pattern and PD-L1 expression in GC, colon cancer and head and neck squamous cell carcinoma [12–14]. However, the underlying regulatory biological process between m6A and lncRNAs in GC, especially their clinical applications in predicting prognosis and immune therapy response remain elusive.

In the present study, we attempted to comprehensively evaluate the correlations of m6A-related lncRNAs with prognosis, immune cell infiltrating level and response to immune checkpoint inhibitors (ICIs) therapy in GC patients. These associations were analyzed multidimensionally, patients with GC were clustered into distinct subtypes characterized by different expression patterns of m6A-related lncRNAs, and then patients were also categorized into high-risk group or low-risk group with the construction of prognostic model. Moreover, our study revealed that m6A-related lncRNAs played a non-negligible role in shaping TIME and predicting responses to ICIs therapy.

## Materials and methods

### Data collection and processing

RNA sequencing data and clinical information were downloaded from the TCGA database via the GDC data portal (https://portal.gdc.cancer.gov/repository) and the raw counts of 375 GC samples and 32 normal samples were collected. Raw counts were converted into transcripts per million (TPM) for subsequent analysis. Raw counts were also transformed to log2 (TPM + 1) when the following analysis was required. Next, we obtained a total of 14,086 lncRNAs according to the Ensemble IDs of the genes for further analysis. Additionally, corresponding clinical information of GC patients in TCGA database were obtained from Liu et al. [15]. Four commonly used clinical outcome endpoints (OS: overall survival, DSS: disease specific survival, DFI: disease-free interval, PFI: progression-free interval) were analyzed. Patients with missing survival status or time information of OS were excluded. Ultimately, 371 GC patients with lncRNA expression data and clinicopathological information including age, gender, grade, stage and TNM staging were selected in the final cohort for analysis. A total of 371 patients were randomly assigned into the training or validation cohort at the ratio of 7:3 using the “caret” R package. The baseline characteristics of the included TCGA dataset were summarized in Table 1. Continuous variables were converted to categorical variables for further analysis. Microsatellite Instability (MSI) status and immunophenoscore (IPS) for each sample in TCGA were downloaded from The Cancer Immunome Database (TCIA) (https://tcia.at/home).

**Table 1:** Clinical characteristics of GC patients in TCGA database

Characteristics		Total TCGA (*N* = 371)
Age (years)	≦65	159
	> 65	208
	unknown	4
Gender	Male	134
	Female	237
Stage	Stage I-II	164
	Stage III-IV	184
	unknown	23
Grade	Grade 1–2	146
	Grade 3	126
	unknown	9
T	T1–2	99
	T3–4	264
	unknown	8
M	M0	327
	M1	26
	unknown	18
N	N0	114
	N1–3	242
	unknown	15

### Identification of the prognostic m6A-related lncRNAs

Based on previous publications, expression matrixes of 23 m6A-related genes were extracted according to the mRNA expression data in TCGA, including expression data on writers (METTL3, METTL14, METTL16, WTAP, VIRMA, ZC3H13, RBM15 and RBM15B), readers (YTHDC1, YTHDC2, YTHDF1, YTHDF2, YTHDF3, HNRNPC, FMR1, LRPPRC, HNRNPA2B1, IGFBP1, IGFBP2, IGFBP3 and RBMX) and erasers (FTO and ALKBH5). Subsequently, m6A-related lncRNAs were first filtered using Pearson correlation analysis with correlation coefficient > 0.4 and *p* < 0.001 based on the transformed TPM values (log2 (TPM + 1)) of 14,086 lncRNAs and 23 m6A-related genes. Co-expression network graph was plotted by the “igraph” R package. Then univariate Cox regression analysis was conducted to screen the prognostic m6A-related lncRNAs with the criterion of false discovery rate (FDR) < 0.05. Wilcoxon test was applied to examine the expression differences of lncRNAs between GC tissue and normal adjacent tissues.

### Identification of m6A-related lncRNAs subgroups by consensus clustering method

To further explore the underlying biological characteristics of the m6A-related lncRNAs, 371 GC patients were clustered into different subtypes using the “ConsensusClusterPlus” R package with iterations of 50 and resample rate of 0.8 based on the transformed TPM data of the selected m6A-related lncRNAs after conducting univariate Cox regression analysis. The optimal k value (k = 2) was determined to obtain stable clusters. Kaplan Meier survival method and log rank test were used for subgroup analysis of clinicopathological factors between two clusters.

### Analysis of the correlations of different clusters with TIME

Scores of immune, stromal and ESTIMATE were calculated using ESTIMATE algorithm by the “estimate” R package. Immune, stromal and ESTIMATE scores were compared between 2 clusters by Wilcoxon test. MIXTURE algorithm with LM22 signature [16] was implemented to estimate the immune infiltrate with TPM value. We conducted Wilcoxon test to compare the abundance of immune infiltrating cells between 2 clusters.

GSEA was conducted to investigate the biological pathways that patients in cluster1 enriched in compared with those in cluster2 with random sampling of 1000 permutations, setting the criteria of FDR < 0.05.

### Construction and validation of the risk model and its association with clinicopathological features and immune infiltrating cells

The least absolute shrinkage and selection operator (LASSO) COX regression algorithm was implemented to further select the m6A-related lncRNAs most associated with OS in the training cohort using the “glmnet” R package. Thereafter, the expression level of the identified lncRNAs and their corresponding coefficients obtained from the LASSO regression algorithm were used to establish the risk model. The risk score calculating formula is:

$$Risk\ score=\sum_{k=1}^n{coef}_k\ast {x}_k$$

Where *Coef*_*k*_ refers to the coefficient of each lncRNA and X_*k*_ refers to the TPM value of each m6A-related lncRNA.

Patients were divided into high-risk group and low-risk group in both training and testing groups according to the median risk score. To evaluate the prognosis prediction accuracy of the risk model, receiver operating characteristic (ROC) was applied in both training and validation cohorts. Kaplan Meier survival method and log rank test were implemented to detect the OS difference between low-risk group and high-risk group. Subsequently, subgroup survival analysis of clinicopathological features were utilized in different risk groups. Univariate and multivariate Cox regression analysis were conducted to evaluate whether the risk score was independent prognostic factor. Wilcoxon test was implemented to further explore the risk score differences with regard to clinicopathological factors, immune score, TMB scores, IPS and two clusters. Pearson correlation test was implemented to evaluate the relationship of risk scores with the abundance of immune infiltrating cells and TMB scores.

### Null distribution analysis

To verify the robustness of classification of GC patients with different prognostic outcomes, we performed additional classifications based on randomly selected genes and tested whether the randomly generated molecular signatures were also correlated with OS of GC patients. A set of 50 genes were extracted from transcript data of GC samples in TCGA, the samples were classified into 2 categories based on principal component analysis (PCA). Finally, we selected a random subset of samples to match the number of samples in classifications based on m6A-related lncRNAs. This process was repeated 5000 times. Hazard ratio and *p* value were generated each time. The detailed method of null distribution analysis can be referred to Rocha et al. [17].

### Calculation of TMB scores

We calculated the mutation frequency with number of variants/the length of exons (38 million) for each sample via Perl scripts based on the JAVA platform. To acquire of somatic mutation data, we downloaded “Masked Somatic Mutation” data and used VarScan software to process it. We implemented “maftools” R package to analyze and visualize the Mutation Annotation Format (MAF) of somatic variants. Wilcoxon test and Pearson correlation analysis were conducted to analyze the relationship between TMB and risk score.

### Construction of the ceRNA network

The miRcode database was used to predict the potential target miRNAs with which the 15 candidate lncRNAs might interact [18]. Thereafter, target mRNAs of these miRNAs were retrieved based on miRTarBase [19], miRDB [20], and TargetScan database [21]. Next, the lncRNA-miRNA-mRNA ceRNA network was established and visualized with the Cytoscape v.3.5.1 [22]. Kyoto Encyclopedia of Genes and Genomes (KEGG) pathway enrichment analysis of the target mRNAs was performed using the “clusterProfiler” R package.

### External validation in clinical samples

We collected tumor samples and adjacent normal samples from 35 GC patients with surgical resection from the Affiliated Hospital of Nantong University. Fresh tumor and adjacent normal tissue were stored at − 80 °C. This research was approved by the Clinical Research Ethics Committee of the Affiliated Hospital of Nantong University (2021-L018). For evaluating the expression level of the selected 8 m6A-related lncRNAs, total RNA from 35 gastric cancer samples and their adjacent normal tissues were extracted by using MolPure Cell RNA Kit (YEASEN Biotech Co., Ltd). cDNA synthesis was carried out by using Hifair III 1st Strand cDNA Synthesis SuperMix for qPCR (YEASEN Biotech Co., Ltd). The relative lncRNA expression levels were calculated with 2^–ΔΔCT^ method, GAPDH was served as an internal control, the sequences of qPCR primers were presented at Table S1. Risk score was calculated by the above constructed formula, median risk score was determined as the cut-off value. Then univariate and multivariate Cox regression analysis, Kaplan Meier analysis, time-dependent ROC curve and calibration curve were conducted for the validation of prognosis value in the external cohort (*N* = 35).

## Results

### Identification of m6-related lncRNAs in GC patients

**Fig. 1:**
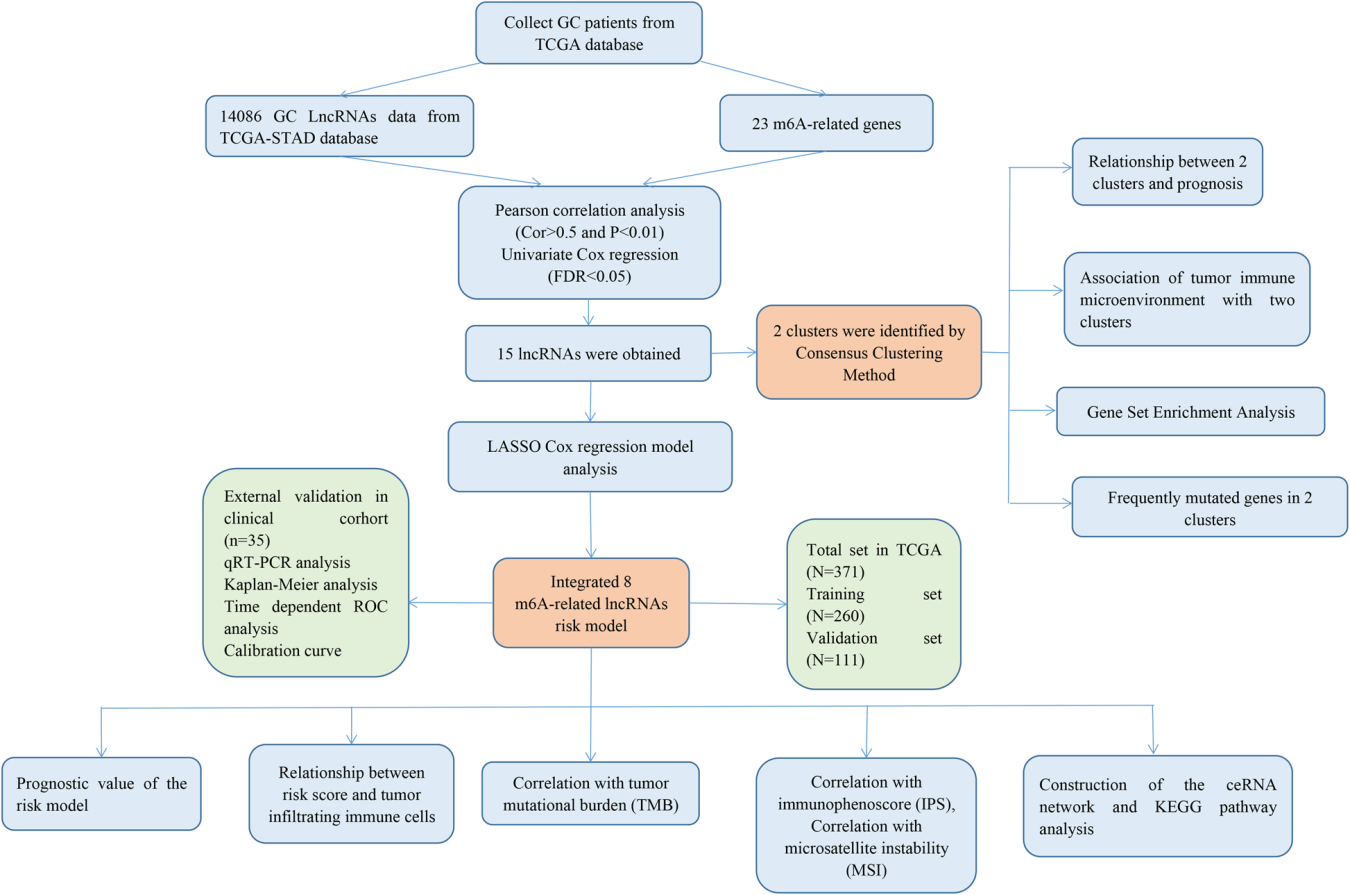
Study flow chart of this study

**Fig. 2:**
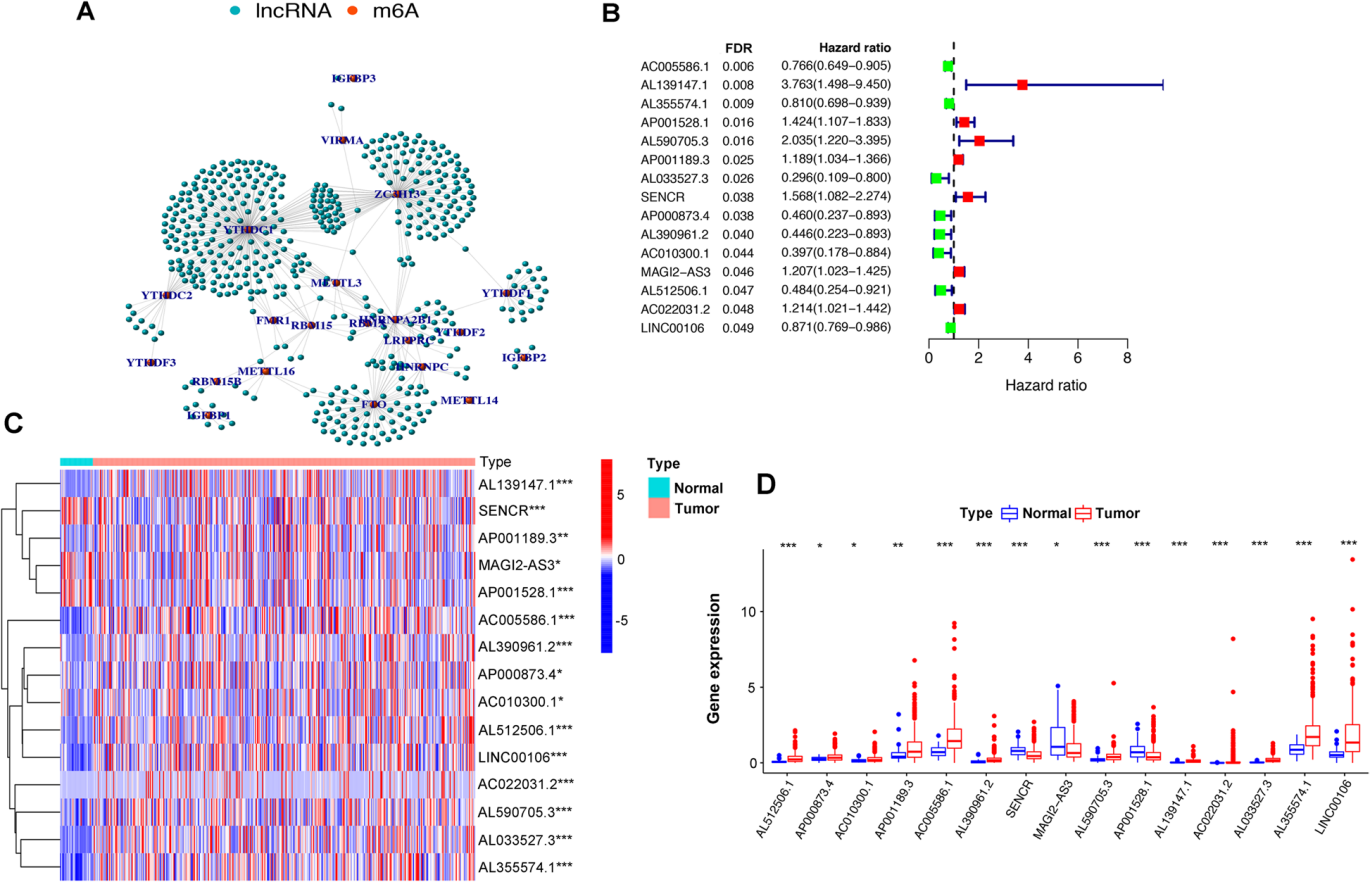
The lncRNAs significantly correlated with m6A-related genes. **A** Co-expression network of m6A-related genes and lncRNAs. **B** Forest plot of the prognostic ability of the 15 m6A-related lncRNAs with FDR < 0.05. **C**, **D** Heatmap (**C**) and expression value (**D**) of the 15 m6A-related lncRNAs in 32 normal tissues and 375 tumor tissues. * *p* < 0.05, ** *p* < 0.01, *** *p* < 0.001. FDR: False Discovery Rate

The study flowchart is shown in Fig. 1. The expression matrixes of a total of 14,086 lncRNAs and 23 m6A-related genes were extracted from TCGA-STAD RNA sequencing dataset. The value of Pearson correlation > 0.4 and *p* value < 0.01 were set as the criterion for preliminarily selecting m6A-related lncRNAs. Four hundred ninety-one lncRNAs were found to be significantly correlated with m6A-related genes. The co-expression network graph was shown in Fig. 2A. Subsequently, 15 lncRNAs were obtained by univariate Cox regression analysis (FDR < 0.05) when the prognostic information was integrated. The forest plot showed that 7 of the screened lncRNAs were risk factors with Hazard Ratio (HR) > 1 and others were protective factors with Hazard Ratio (HR) < 1 (Fig. 2B). The heatmap (Fig. 2C) and boxplot (Fig. 2D) presented the 15 lncRNAs expression pattern in the GC tissues and normal tissues.

### Consensus clustering identified two clusters of GC samples with distinct prognoses based on m6A-related lncRNAs

**Fig. 3:**
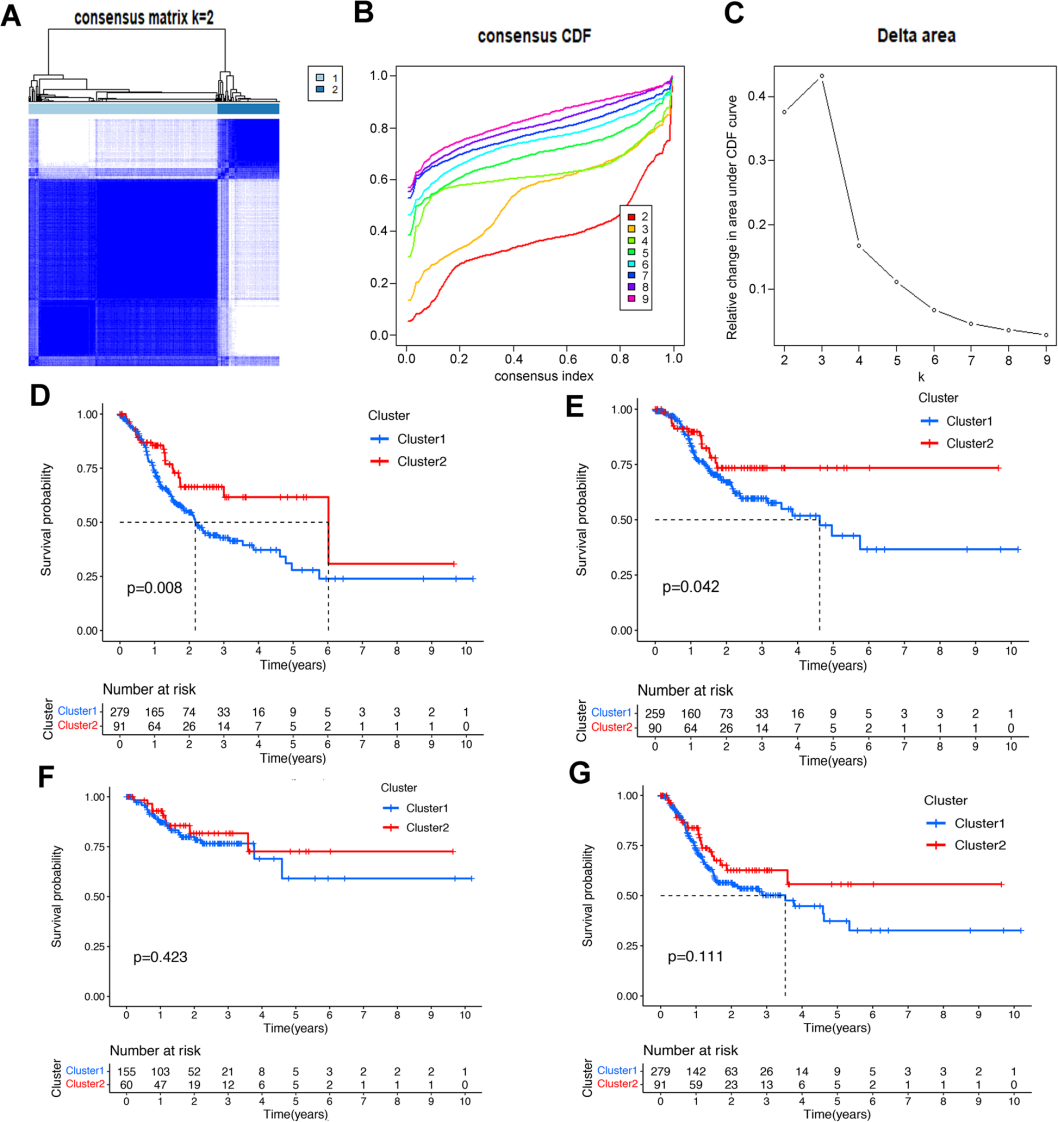
Differential survival outcomes of GC in cluster 1/2 subtypes. **A** Consensus score matrix of all samples when k = 2. **B** The cumulative distribution functions (CDF) for k = 2 to 9. **C** Relative change in area under CDF area for k = 2 to 9. **D-G** Kaplan-Meier curves for OS (**D**), DSS (**E**), DFI (**F**) and PFI (**G**) in cluster 1/2 subtypes. OS: overall survival, DSS: disease specific survival, DFI: disease-free interval, PFI: progression-free interval

Based on the similarity of the expression of the 15 lncRNAs in GC samples, consensus clustering method was applied to cluster the samples to further elucidate the biological discrepancies among subgroups. The CDF curves of consensus matrix indicated that when k = 2, the interference between subgroups is minimal and the distinction is significant (Fig. 3A-C, Fig. S1A-G). A total of 371 GC patients were separated into cluster1 (*n* = 266) and cluster2 (*n* = 105). As illustrated in the survival plots, patients in cluster1 had worse OS and DSS than those in cluster2 did (Fig. 3D-E), while no significant differences were observed in DFI and PFI between two subgroups (Fig. 3F-G).

The association of the two clusters and GC patients’ prognoses was further explored by comparing the OS of the two clusters in different clinical subgroups. The survival plots were drawn (Fig. S2A-G) and the results showed that the OS of 2 clusters had significant differences in age >  65 (*p* < 0.001), female (*p* = 0.003), G1–2 (*p* = 0.003), M0 (*p* = 0.010), N1–3 (*p* = 0.043), stage III-IV (*p* = 0.038) and T3–4 (*p* = 0.012). More importantly, the consensus clustering method performed better than 83% of the classifications based on random sets of genes, suggesting that the classification based on m6A-related lncRNAs could serve as a robust prognostic indicator.

### Tumor immune microenvironment (TIME) and mutation profile of two clusters

**Fig. 4:**
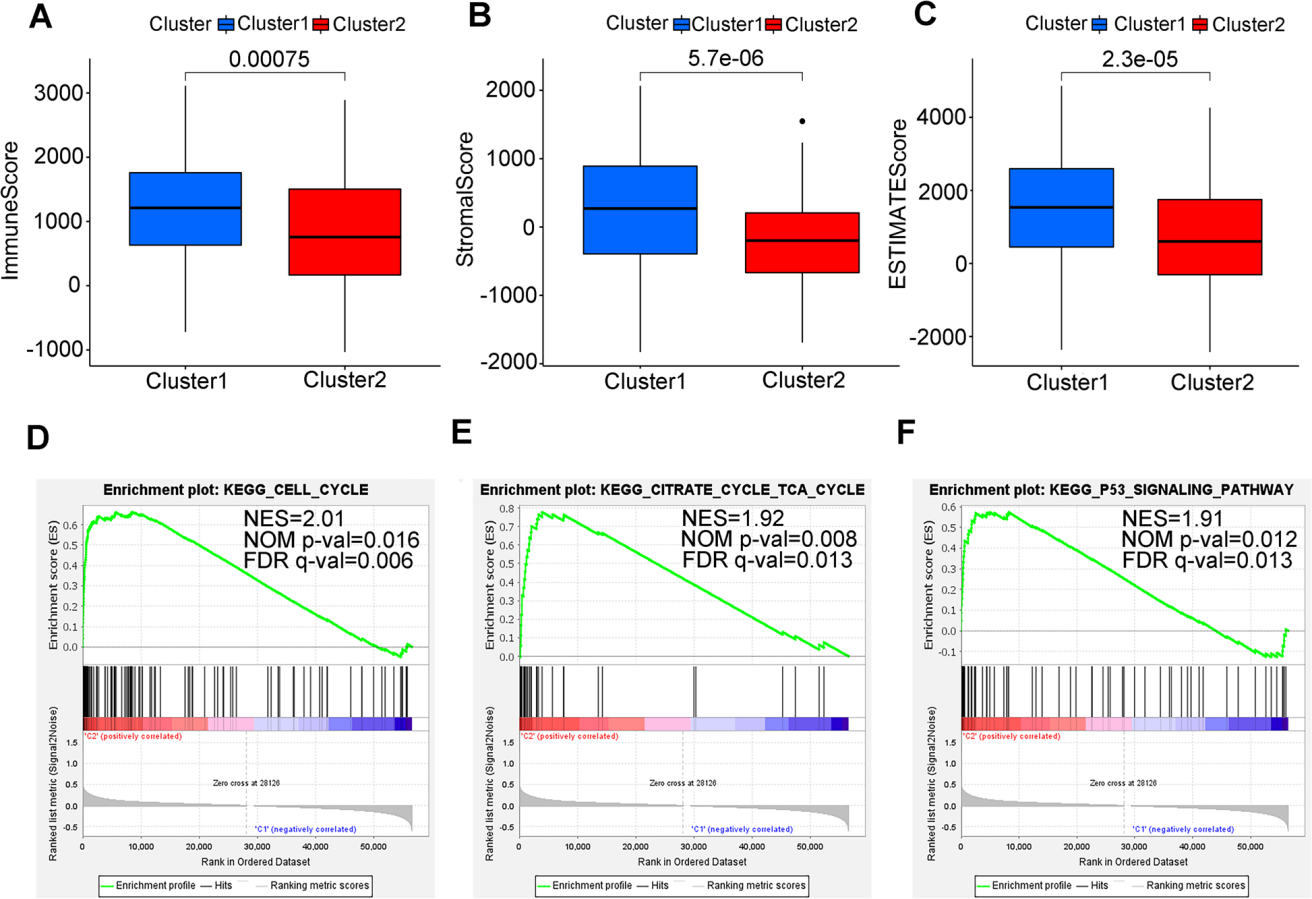
ESTIMATE analysis and GESA in cluster 1/2 subtypes. **A-C** Comparison of immune score (**A**), stromal score (**B**) and ESTIMATE score (**C**) in two subtypes. **D-F** GSEA showed that cell cycle, citrate cycle (TCA cycle) and P53 signaling pathway are differentially enriched in cluster1. NES, normalized enrichment score; NOM *p*-val, normalized *p* value; FDR q value: False Discovery Rate q value

TIME as a crucial cellular milieu for immune cells, stromal cells and extracellular matrix molecules has predominant impact on the tumor progression. To get deeper insights into the interplay between m6A-related lncRNAs and immune activity, the distribution differences of the estimated proportion of immune and stromal between the 2 clusters were calculated by ESTIMATE algorithm. As shown in Fig. 4A-C, cluster1 harbored higher immune score (*p* < 0.001), stromal score (*p* < 0.001) and ESTIMATE score (*p* < 0.001). These findings revealed that larger amount of immune and stromal components in TIME of GC were associated with worse survival outcome.

For investigating the biological mechanism of contributing the heterogeneity of the two clusters, GSEA was implemented. We found that several tumor hallmarks were dynamically enriched in the cluster1 compared with cluster2, including cell cycle pathway (normalized enrichment score [NES] = 2.01, FDR q value = 0.006), citrate cycle (TCA cycle) pathway (NES = 2.18, FDR q value = 0.013), and P53 signaling pathway (NES = 1.91, FDR q value = 0.001) (Fig. 4D-F) [23–25]. Aforementioned multiple signaling differences between the two clusters indicated the potential role of m6A-related lncRNAs in gastric carcinogenesis.

**Fig. 5:**
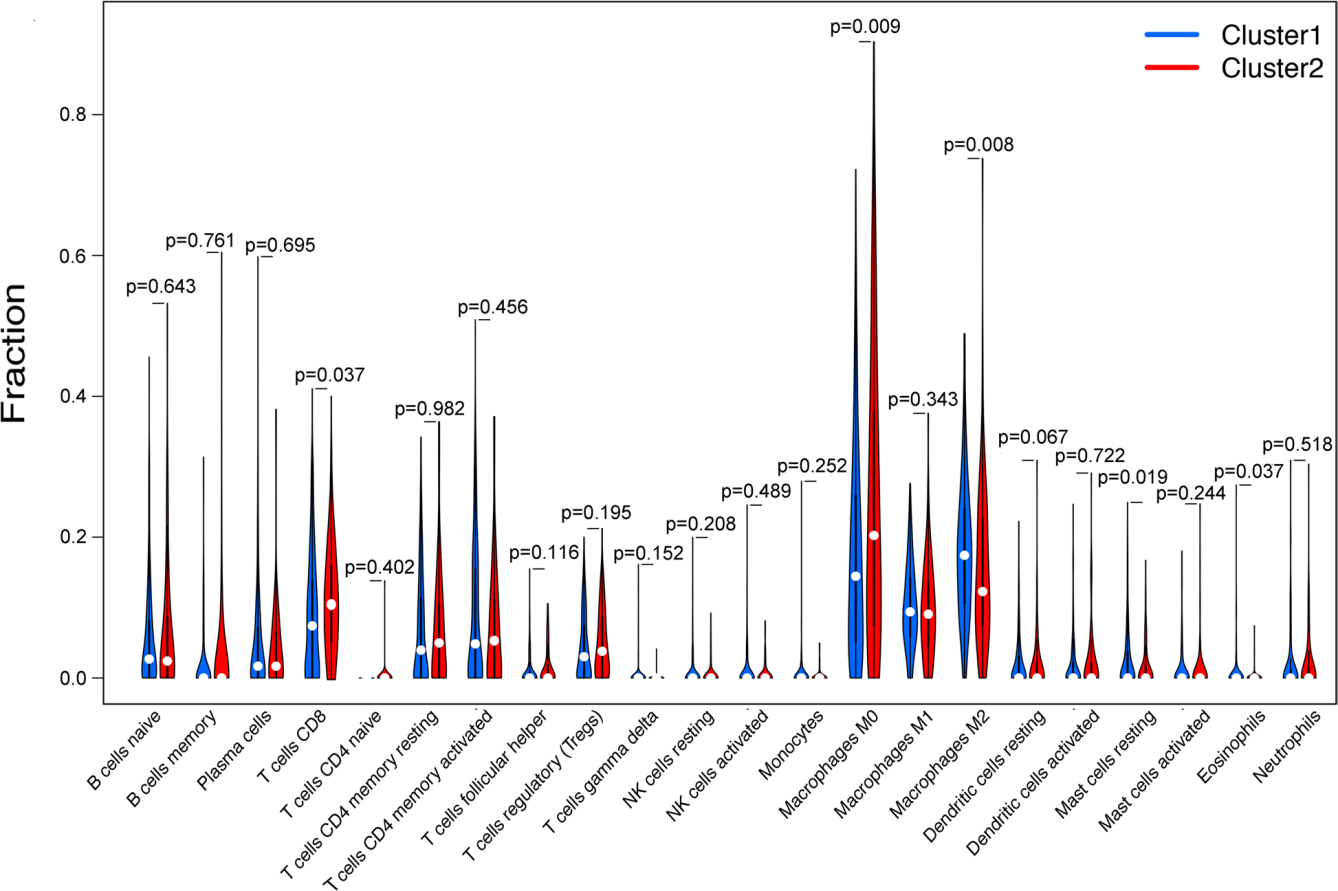
The fraction of 22 types of infiltrating immune cells in cluster 1/2 subtypes

**Fig. 6:**
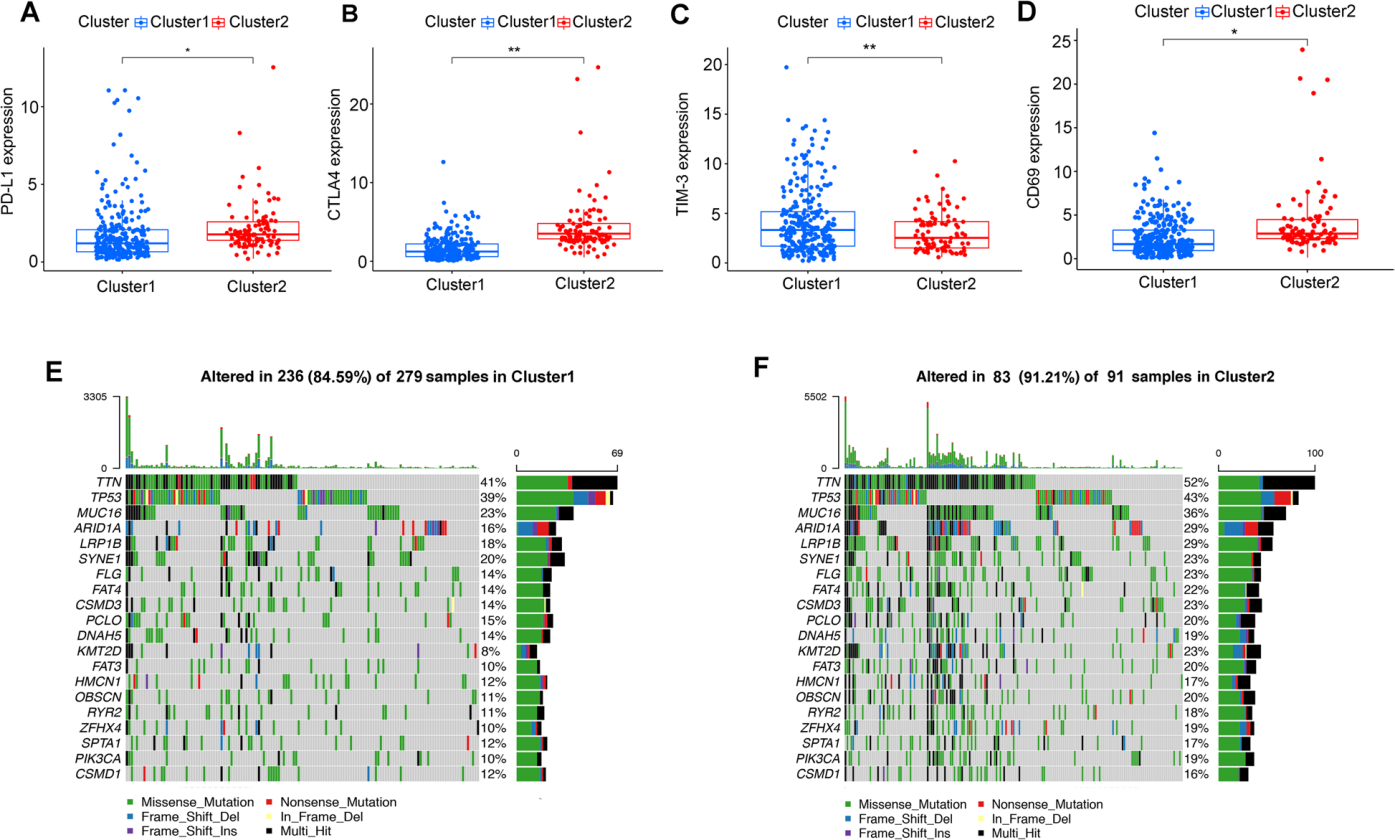
Frequently mutated genes and expression of immune related genes in cluster 1/2 subtypes. **A-D** The gene expression pattern of PD1, CTLA4, TIM-3 and CD69 in cluster 1/2. **E**, **F** Waterfall plots display the frequently mutated genes in 2 clusters of gastric cancer. The left panel shows the genes ordered by their mutation frequencies. The right panel shows different mutation types

To elucidate the specific immune cells distribution pattern in two subtypes, the fraction of 22 immune cell types were analyzed by MIXTURE algorithm (Fig. 5). We discovered that cluster2 enriched in CD8^+^ T cells (*p* = 0.037) and M0 macrophages (*p* = 0.009). Conversely, cluster1 enriched in M2 macrophages (*p* = 0.008), resting mast cells (*p* = 0.019) and eosinophils (*p* = 0.037). Furthermore, cluster2 also exhibited higher expression level of PD-L1 (*p* < 0.05) (Fig. 6A) and CTLA4 (*p* < 0.01) (Fig. 6B) than cluster1. CD69 acts as a costimulatory molecule for T cell activation and proliferation while TIM-3 has the opposite function. Therefore, we further explored the status of T cell in different clusters, and found that cluster1 had higher expression level of TIM-3 (*p* < 0.01) (Fig. 6C) but lower expression level of CD69 (*p* < 0.001) (Fig. 6D), indicating that the status of CD8 T cell was probably more active in cluster2 than that in cluster1.

Finally, we evaluated the association between mutation profiles and two clusters. The mutation profiles of each sample in 2 clusters (cluster1: 84.59%, cluster2: 91.21%) were presented respectively (Fig. 6E and F). We found that the mutated genes had substantially different frequencies in two clusters.

### Construction of the ceRNA network and functional enrichment analysis

**Fig. 7:**
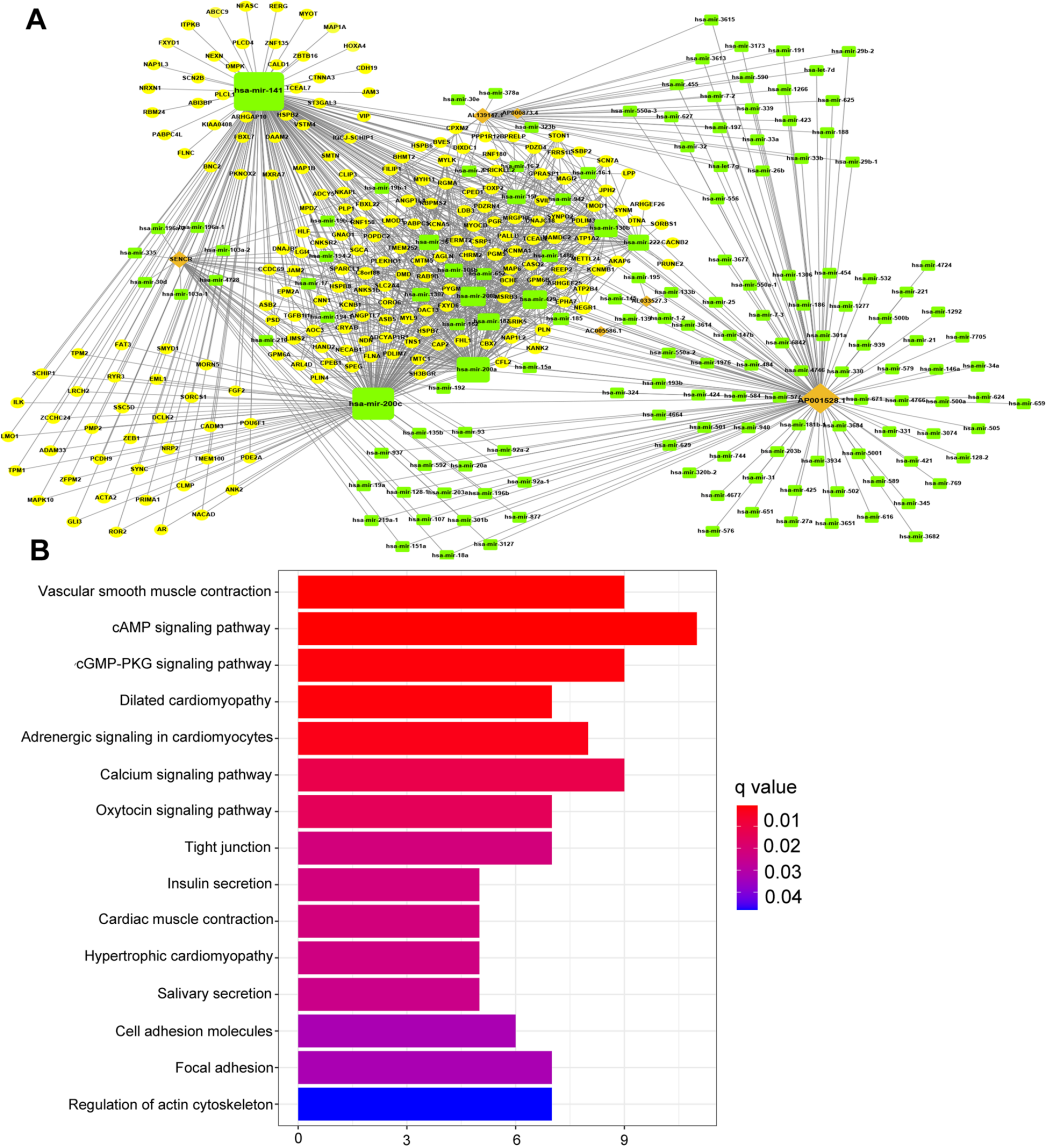
Construction of ceRNA network. **A** The ceRNA network of the six m6A-related lncRNAs (orange) and their target miRNAs (green) and mRNAs (blue). **B** KEGG pathway enrichment of target mRNAs

We attempted to further explore the underlying biological processes the 15 candidate m6A-related lncRNAs might take part in by sponging miRNAs and regulating mRNA expression. First, we extracted 6 m6A-related lncRNAs (SENCR, AL139147.1, AP000873.4, AC005586.1, and AL033527.3) from miRcode database and 221 pairs of interaction between the 6 lncRNAs and 152 miRNAs were identified. Then, we searched three databases (TargetScan, miRDB, and miRTarBase) to found target mRNAs based on the identified 152 miRNAs, and 209 mRNAs were included consequently. The ceRNA network among the 6 lncRNAs, 152 miRNAs, and 209 mRNAs was constructed accordingly (Fig. 7A). Finally, the 209 mRNAs were selected to conduct KEGG enrichment analysis. The results of KEGG analysis demonstrated that the m6A-related lncRNAs in GC mainly enriched in vascular smooth muscle contraction, cAMP signaling pathway and cGMP-PKG signaling pathway (Fig. 7B) [23–25].

### Construction and validation of a risk score model for GC patients based on m6A-related lncRNAs

**Fig. 8:**
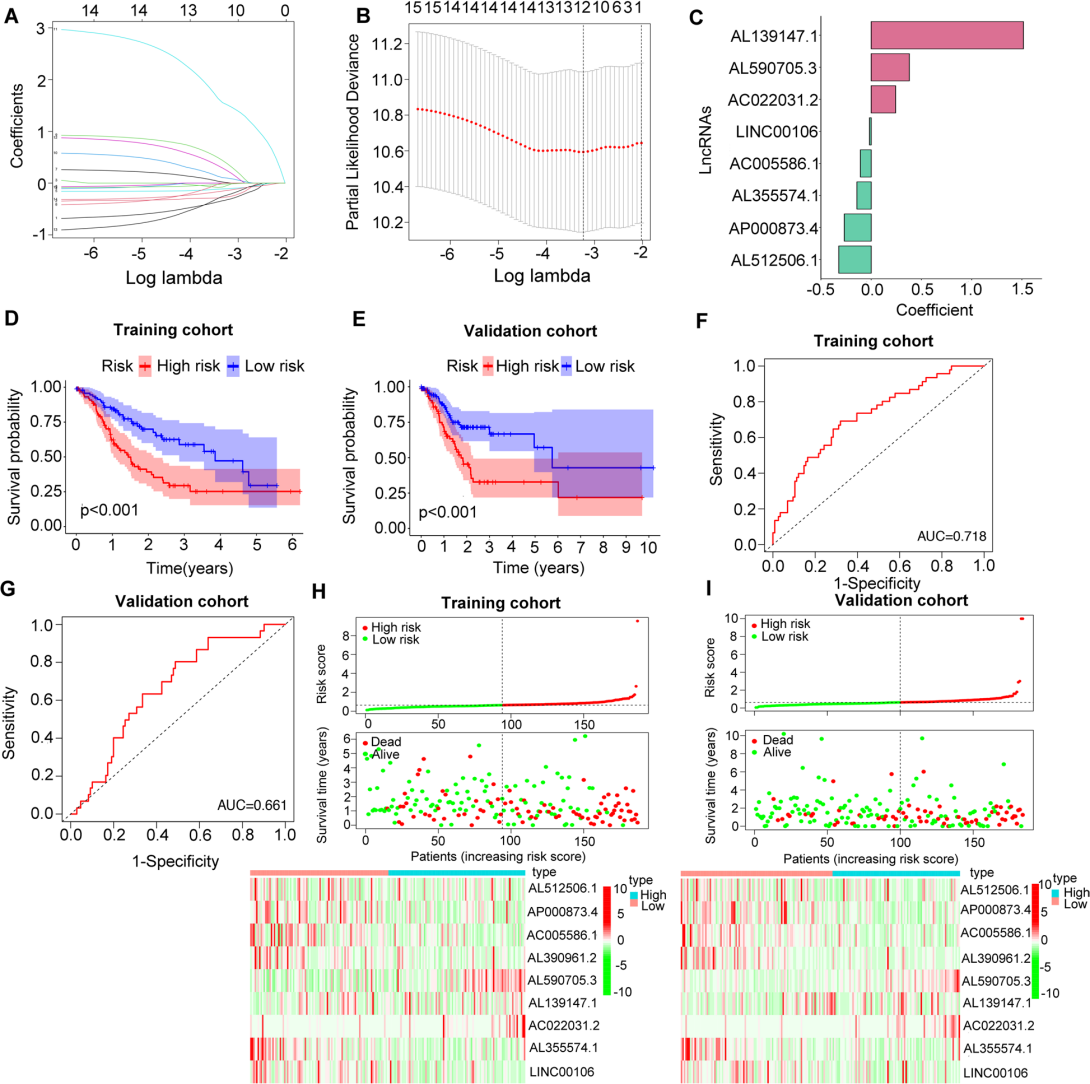
Construction of the GC prognostic risk model based on 8 m6A-related lncRNAs. **A**, **B** Least absolute shrinkage and selection operator (LASSO) regression was performed, the penalization coefficient λ in the LASSO model was tuned using 10-fold cross-validation and minimum criterion for the selection of m6A-related lncRNAs. **C** Barplot of the coefficients of selected lncRNAs. **D**, **E** Kaplan Meier analysis showed that high-risk group exhibited worse survival outcome than low-risk group in the training (**D**) and validation cohort (**E**). **F**, **G** Receiver operating characteristic (ROC) curves of risk model for predicting survival in the training (**F**) and validation cohort (**G**). **H**, **I** Distribution of risk score, survival statuses of GC patients and expression levels of the 8 m6A-related lncRNAs in high-risk group and low-risk group in the training (**H**) and validation cohort (**I**)

As assessed by the univariate Cox regression analysis, 15 differentially expressed m6A-related lncRNAs were found significantly associated with OS. To identify the most powerful prognostic m6A-related lncRNAs, LASSO regression analysis was performed. A total of 8 lncRNAs containing AL139147.1, AL590705.3, AC022031.2, LINC00106, AC005586.1, AL355574.1, AP000873.4 and AL512506.1 were finally identified (Fig. 8A-C). Based on the regression coefficients and expression values of the 8 lncRNAs, a risk model was constructed. The following formula was present: risk score = 1.515 * expression (AL139147.1) + 0.380 * expression (AL590705.3) + 0.243 * expression (AC022031.2) - 0.017 * expression (LINC00106) – 0.107 * expression (AC005586.1) - 0.142 * expression (AL355574.1) - 0.267 * expression (AP000873.4) – 0.322 * expression (AL512506.1). According to the median value of the risk score. GC patients were divided into high-risk group and low-risk group. We found that both in training cohort and validation cohort, GC patients from high-risk group had worse survival outcome (*P* < 0.001) (Fig. 8D and E). The ROC curves were utilized to evaluate the prognosis prediction performance of risk model, and the area under the ROC curve (AUC) for OS was 0.718 in the training cohort (Fig. 8F) and 0.661 (Fig. 8G) in the validation cohort. Moreover, the distribution of risk scores and survival statuses of patients in the both training and validation cohort were displayed in Fig. 8H and I. These figures indicated that with the increase of risk score, the mortality rate was increased and the survival time was decreased. The heatmap presented the distinct expression patterns of the 8 lncRNAs between different risk groups. Taken together, our results suggested that the risk scores based on the 8 m6A-related lncRNAs had optimal prediction ability of the prognosis of GC patients.

### Risk score based on the m6A-related lncRNAs was an independent prognostic factor for GC patients

We performed subgroup analyses for GC patients with different risk groups from different subgroups stratified by age, gender, grade or stage, most of subgroups had significant survival differences in different risk groups (Fig. S3A-L). Then we performed univariate and multivariate Cox regression analysis to determine whether the risk model based on the m6A-related lncRNAs could independently predict the prognosis of GC patients. The results of univariate Cox regression analysis showed that OS was evidently associated with age, grade, stage and risk score in the training cohort, and OS was significantly associated with stage and risk score in the validation cohort. After adjusting the effects of clinicopathological factors such as age, gender, grade and stage in the multivariate Cox regression analysis, age (HR, 1.058; 95%CI, 1.031–1.086, *p* < 0.001), grade (HR, 1.754; 95%CI, 1.080–2.846, *p* = 0.023) and stage (HR, 1.483; 95%CI, 1.085–2.027, *p* = 0.014) and risk score (HR, 1.798; 95%CI, 1.484–2.179, *p* < 0.001) acted as the powerful prognostic factors in the training cohort, and stage (HR, 1.813; 95%CI, 1.281–2.565, *p* < 0.001) and risk score (HR, 1.872; 95%CI, 1.723–2.098, *p* = 0.04) acted as the powerful prognostic factors (Fig. S4A-D) (Table 2) in the validation cohort.

**Table 2:** Univariate and multivariate analysis of risk score and clinical factors with OS in the training cohort and validation cohort of TCGA and external validation cohort

Risk factor	Training cohort in TCGA (*N* = 260)				Validation cohort in TCGA (*N* = 111)				External cohort (*N* = 35)			
	Univariate analysis		Multivariate analysis		Univariate analysis		Multivariate analysis		Univariate analysis		Multivariate analysis	
	Hazard ratio (95% CI)	*P*-value	Hazard ratio (95% CI)	*P*-value	Hazard ratio (95% CI)	*P*-value	Hazard ratio (95% CI)	*P*-value	Hazard ratio (95% CI)	*P*-value	Hazard ratio (95% CI)	*P*-value
Age	1.048 (1.021–1.075)	< 0.001	1.058 (1.031–1.086)	< 0.001	1.002 (0.977–1.028)	0.890	1.013 (0.985–1.042)	0.360	1.214 (0.321–2.312)	0.235	1.032 (0.231–2.913)	0.451
Gender	1.240 (0.772–1.993)	0.374	1.411 (0.858–2.319)	0.175	1.304 (0.728–2.335)	0.371	1.202 (0.667–2.169)	0.540	1.152 (0.489–2.182)	0.552	0.924 (0.451–3.521)	0.461
Grade	1.638 (1.031–2.601)	0.037	1.754 (1.080–2.846)	0.023	1.117 (0.676–1.845)	0.665	1.104 (0.655–1.861)	0.711	3.456 (1.224–5.050)	0.024	2.341 (0.219–7.121)	0.034
Stage	1.457 (1.099–1.933)	0.009	1.483 (1.085–2.027)	0.014	1.671 (1.209–2.308)	0.002	1.813 (1.281–2.565)	< 0.001	2.341 (0.512–5.641)	0.235	1.425 (0.248–7.214)	0.810
riskScore	1.674 (1.395–2.008)	< 0.001	1.798 (1.484–2.179)	< 0.001	1.513 (1.102–2.731)	0.007	1.872 (1.723–2.098)	0.04	2.424 (1.516–3.124)	0.013	1.915 (1.529–2.942)	0.040

### Prognostic risk score was associated with clinicopathological factors and immune infiltrating cells

**Fig. 9:**
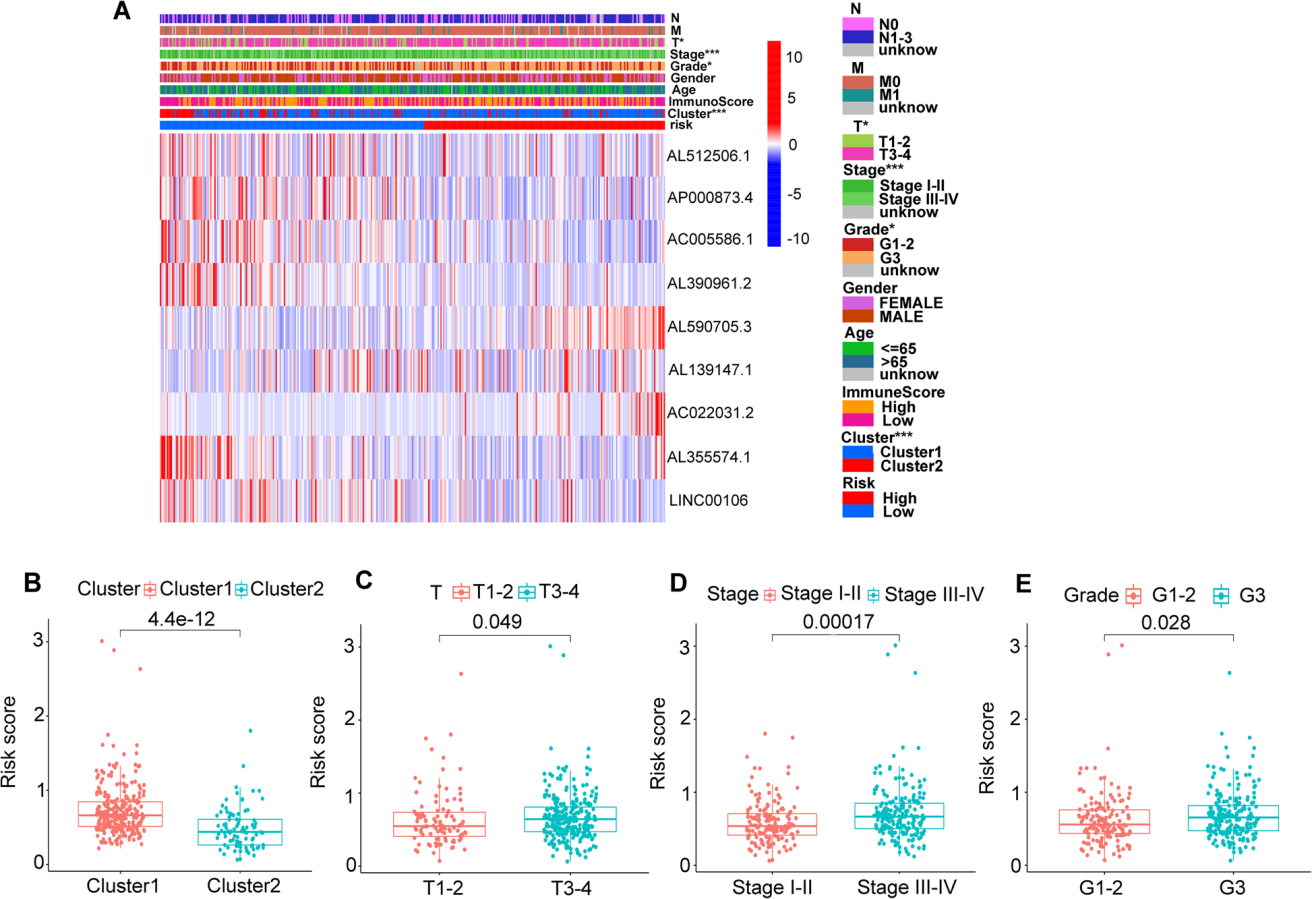
Prognostic risk score correlated with clinicopathological features and cluster1/2 subtype. **A** Heatmap of clinicopathological features, immune score and different lncRNAs expression pattern in high/low risk group. **B-E** Comparison of risk score distribution in the cluster1/2 (**B**), T1–2/T3–4 (**C**), stage I-II/III-IV (**D**) and G1–2/G3 (**E**). * *p* < 0.05, *** *p* < 0.001. G1–2: Grade1–2, G3: Grade3

First off, we further evaluated the relationship between risk score and the 8 prognostic m6A-related lncRNAs. As expected, it could be concluded from the heatmap that all the 8 prognostic m6A-related lncRNAs had relatively different expression values in patients from different risk groups (Fig. 9A). Next, it can be also referred from the heatmap and the scatter diagrams (Fig. 9B-E) that statistical differences of risk scores existed in the different clusters (*p* < 0.001), T1–2 vs T3–4 (*p* = 0.049), stage I-II vs stage III-IV (*p* < 0.001) and grade1 + grade2 vs grade3 (*p* = 0.028). Collectively, these facts strongly indicated that risk score established on m6A-related lncRNAs exhibited valuable clinical information and had crucial clinical implication values.

### Risk score was associated with biomarkers for predicting response to ICIs therapy

**Fig. 10:**
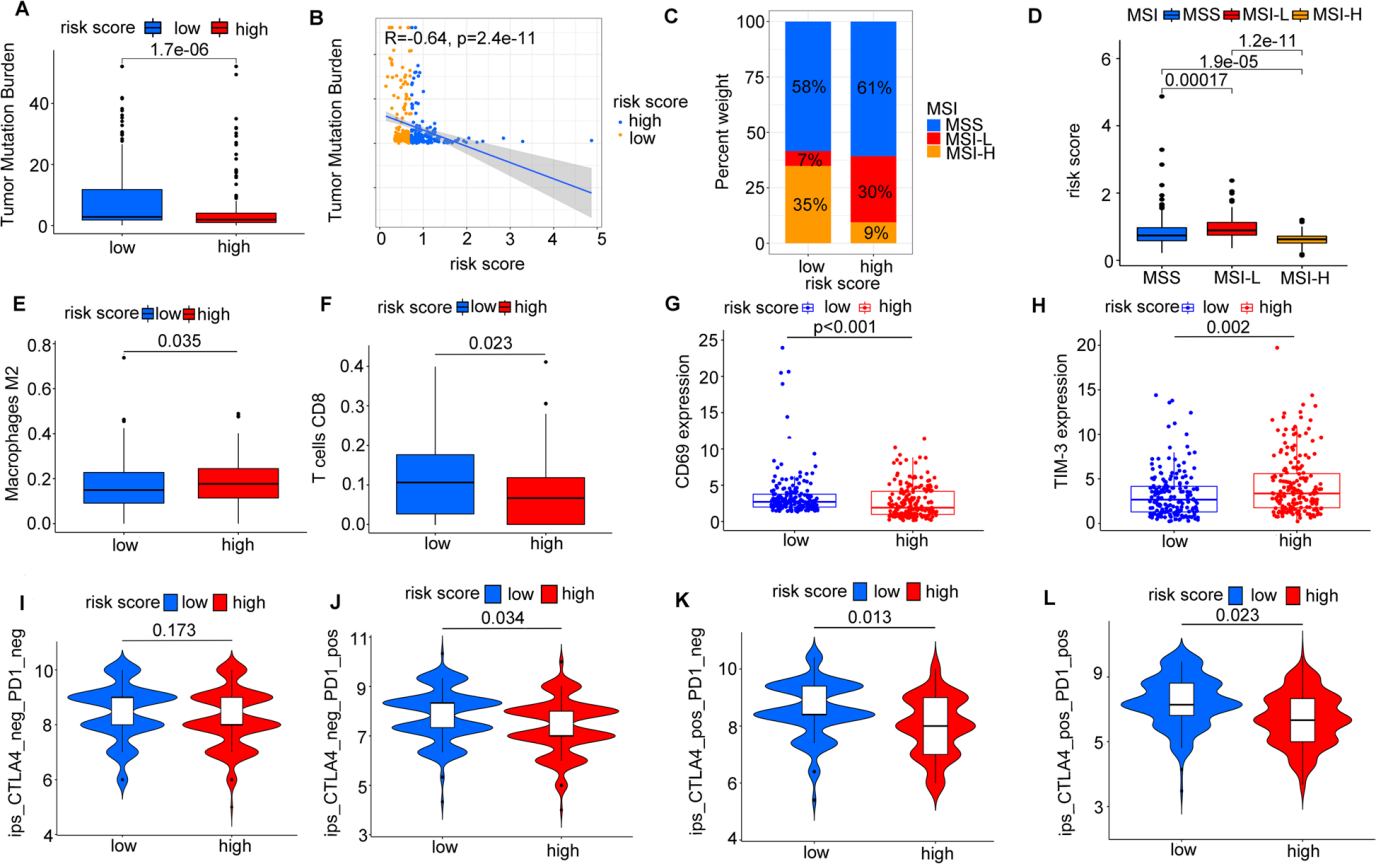
The associations between risk score and immunotherapy. **A** The expression levels of tumor mutation burden in low-risk group and high-risk group. **B** The correlation of risk score and tumor mutation burden. **C** The distribution of microsatellite status in different risk groups. **D** The risk score in patients with different microsatellite statuses. **E-F** The abundance of M2 macrophages infiltration and CD8 T cells infiltration in high-risk group and low-risk group. **G-H** The expression pattern of CD69 and TIM-3 in high-risk group and low-risk group. **I-L** The relationship between different risk groups and immunophenoscores

We attempted to further excavate the value of the risk model constructed based on the 8 m6A-related lncRNAs in predicting patients’ immunotherapeutic outcomes. Higher TMB was characterized by favorable responses to ICIs therapy. In our study, high TMB was correlated with lower risk score calculated by Wilcox test (*p* < 0.01) (Fig. 10A). Risk score was negatively associated with TMB (R = − 0.84, *p* < 0.001) (Fig. 10B). We also analyzed the distribution differences of MSI between low-risk group and high-risk group. As presented in Fig. 10C, low-risk group showed higher proportion of MSI-H (35% vs 9%) and lower proportion of MSS (58% vs 61%) and MSI-L (7% vs 30%) compared with high-risk group (Fig. 10C). Similarly, MSI-H group exhibited markedly lower risk score than MSI-L group (*p* < 0.001) and MSS group (*p* < 0.001). The risk score of MSI-L group was statistically higher than that of MSS group (*p* < 0.001) (Fig. 10D). We also observed that high-risk group was positively associated with the abundance of M2 macrophages (*p* = 0.035) and negatively associated with abundance of CD8 T cells (*p* = 0.023) (Fig. 10E-F). Patients from low-risk group tended to have active immune status with significantly higher CD69 expression (*p* < 0.001) and lower TIM-3 expression (*p* = 0.002) than those from high-risk group (Fig. 10G-H).

Immunophenoscore was a predictor of response to anti-cytotoxic T lymphocyte antigen-4 (CTLA-4) and anti-programmed cell death protein 1 (anti-PD-1), the relationship between risk score and immunophenoscore was explored. The results showed that different risk groups tended to have different ICIs therapies (anti-CTLA-4 therapy, anti-PD-1 therapy or combination of both therapy) responses (Fig. 10I-L). Our findings strongly indicated that m6A-related lncRNAs had superior values in optimizing patients selection for ICIs therapy and predicting patients’ outcomes of ICI therapy.

### External validation of the 8 m6A-related lncRNAs based risk model in the clinical dataset

**Fig. 11:**
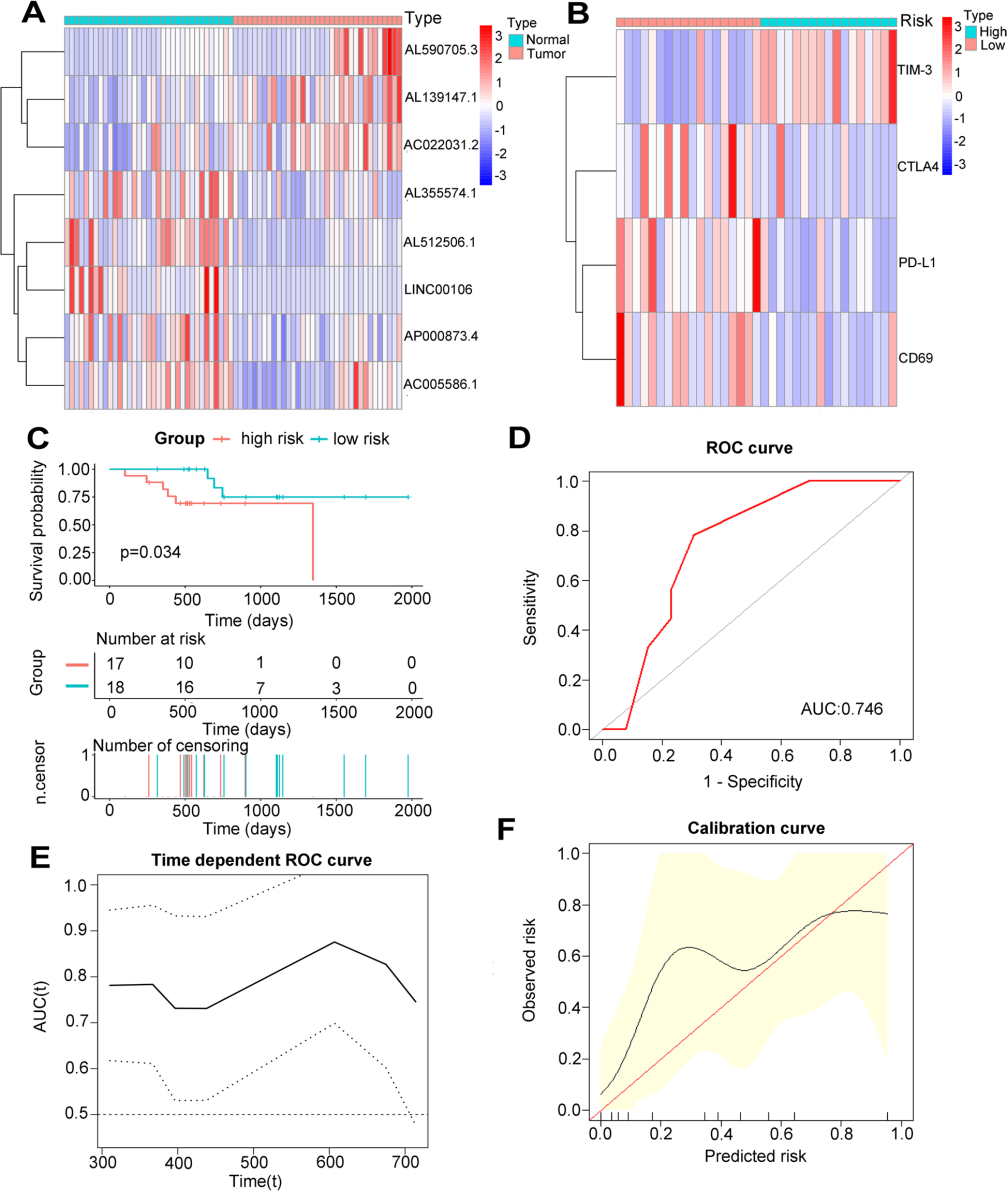
External validation the expression levels of the 8 m6A-related lncRNAs and their prognostic values in 35 GC patients. **A** The heatmap showed the expression pattern of 8 m6A-related lncRNAs in 35 GC tissues and adjacent normal tissues. **B** The heatmap showed the expression pattern of immune related genes in different risk groups. **C** Survival analysis of 35 GC patients in different risk groups. **D** Receiver operating characteristic (ROC) curves of risk model for predicting survival in 35 GC patients. **E** Time dependent AUC analysis where the solid and dashed lines depict the AUC and its 95% CI respectively. **F** The calibration curves of risk score for the prediction of survival

We collected 35 GC patients from the Affiliated Hospital of Nantong University as the external validation cohort. First, we quantitated the relative expression levels of the 8 lncRNAs in 35 tumor tissues and adjacent normal tissues by quantitative RT-PCR, the results demonstrated that the expression pattern of the 8 lncRNAs in our cohort was consistent with TCGA dataset. Three lncRNAs (AL139147.1, AL590705.3 and AC022031.2) were generally up-regulated in tumor tissues, while the other five lncRNAs (AL355574.1, AL512506.1, LINC00106, AP000873.4 and AC005586.1) were mainly down-regulated in tumor tissues (Fig. 11A). Based on the expression level of each lncRNA, we calculated the risk score of each patient and divided total cohort into high-risk group and low-risk group using median risk score as the cutoff value and the baseline clinical information of patients from different risk groups were displayed in the Table 3. We found that TIM-3 was highly expressed in the high-risk group, while CTLA4, PD-L1 and CD69 had lower expression levels in the high-risk group (Fig. 11B). Patients from high-risk group had worse OS compared with those from low-risk group (*p* = 0.034) (Fig. 11C). Univariate and multivariate cox analysis confirmed that the risk score was an independent prognosis predictor (Table 2). To further identify the prognostic value of the risk model, the ROC of the risk score for predicting OS of the external clinical cohort was 0.746 (Fig. 11D). Additionally, time-dependent ROC analysis was applied, and the AUC ranged from 0.731 to 0.876 (Fig. 11E), demonstrating a satisfactory prognostic value. Finally, the calibration curve for the risk score showed good agreement between prediction and actual OS status (Fig. 11F).

**Table 3:** Clinical characteristics of patients in external cohort stratified by risk status

Characteristics	High risk (*n* = 17)	Low risk (*n* = 18)	*P* value
Age			0.642
≦65	6	9	
> 65	9	11	
Gender			0.862
Male	7	10	
Female	10	8	
Stage			0.021
Stage I	2	6	
Stage II	4	7	
Stage III	6	3	
Stage IV	5	2	
Grade			0.034
Grade 1–2	2	8	
Grade 3	7	7	
Grade 4	8	3	
Risk score	5.3 + 1.7	2.5 + 1.2	< 0.001

*P* value for categorical variable was calculated by χ2 test or Fisher’s exact test, for continuous variable, *P* value was calculated by t-test

## Discussion

The interaction pattern between m6A and lncRNAs can be separated into two subtypes. On one hand, m6A modification on lncRNA acted as a structural switch to facilitate RNA-protein interactions. M6A could also mediate the function of lncRNAs by increasing the stability of the transcript of lncRNAs via ceRNA model [7, 26]. For instance, HNRNPC could bind to MALAT1, which was a conserved lncRNA whose upregulation was correlated with carcinogenesis, thereby influencing RNA expression and alternative splicing [27]. On the other hand, IncRNAs could also regulate the m6A regulatory proteins to promote their functions. It was recently reported that in glioblastoma stem-like cells (GSCs), ALKBH5 as a m6A eraser could demethlyse FOXM1 nascent transcripts and enhanced FOXM1 expression. Meanwhile, the FOXM1-AS which was a lncRNA antisense to FOXM1 acted as a promoter for the interaction of ALKBH5 and FOXM1, contributing to the overly expressed FOXM1 and GSC proliferation [28]. Collectively, the sophisticated regulatory network between m6A and lncRNAs provides new possibility in exploring the biological features of carcinogenesis and clinical application roles. In our study, we found that the m6A-related lncRNAs were closely associated with the prognosis of gastric cancer through participating in multiple signaling pathways. Among these signaling pathways, cAMP and cGMP-PKG signaling pathways were reported to be aberrantly regulated in the multiple cancers [29–32]. A recent study found that FTO as a m6A regulator could inhibit the self-renewal of ovarian cancer stem cell through blocking cAMP pathway [33]. cAMP is also known to suppress the T-cell immune function [34], which may explain the mechanism of m6A modification in shaping the immune environment of GC. Our results provide new insights that m6A modification may mainly modulate cAMP and cGMP-PKG signaling pathways in the tumorogenesis of gastric cancer through the interaction between m6A and lncRNAs. The exploration of epigenetic reprogramming in gastric cancer gives us some clues for designing new drugs.

We additionally explored the relationships of m6A-related lncRNAs and clinical characteristics in GC patients, indicating that two subtypes characterized by distinct expression patterns of lncRNAs had significantly different OS outcomes. Besides, the risk score constructed by 8 prognostic m6A-related lncRNAs were also remarkably related with OS outcome. In this regard, it’s not surprising to find that cluster 1 were correlated with high-risk group. This demonstrated that the risk model constructed based on m6A-related lncRNAs was a robust and reliable GC clinical indicator. More importantly, the risk score model was satisfactorily validated in the external cohort, which confirmed its prognostic value.

Several studies have revealed that m6A modification had diverse functions in shaping TIME. YTHDF1 depletion in dendritic cells could enhance the cross-presentation of tumor antigens and cross-priming of CD8+ T cells [35]. In melanoma, FTO could not only promote tumor progression but also lead to anti-PD1 resistance. LncRNAs are also crucial in mediating the development of diverse immune cells [36]. For example, LncRNA UCA1 elevated the expression of PD-L1 via direct interaction with miRNAs in GC [37]. Recently, a new method called ImmLnc pipeline was invented to identify critical lncRNAs involved in TIME, and this method exhibited satisfactory performance in non-small cell lung cancer [38]. Together, although many studies have indicated the separate biological function of m6A or lncRNA enrolling in the TIME, the integrated analysis of regulatory network of them in TIME is still obscure. In our study, we divided GC samples from TCGA into two classes based on the expression pattern of m6A-related lncRNAs. Despite high immune score usually being associated with better survival outcomes in multiple cancer types [39, 40], the subpopulation we identified here showed the opposite trend. This contradictory result may be explained by the varying characteristics of immune cells. The cluster2 we identified here had higher CD8 T cell infiltration and M0 macrophages infiltration, more intriguingly, we also found that cluster2 had more activated T cells status. In this regard, cluster2 might be more sensitive to immunotherapy.

Immunotherapy has revolutionized the oncology landscape, especially the ICIs therapy has gained enormous success in multiple solid tumors [41]. However, only a subset of GC patients can benefit from this novel therapy due to the heterogeneity of immune microenvironment [42]. Therefore, comprehensive analysis of TIME is mandatory for recruiting patients for ICIs therapy. TIME plays a pivotal role in mediating tumor progression by inducing epithelial mesenchymal transition (EMT) in the tumor cells. Stromal cells in the surrounding environment are recruited to the area where the tumor cells localize and promote the distant metastasis [11, 43]. Previous studies investigated the correlation of immune cells in the TIME with GC prognosis. They found that memory T cells, cytotoxic CD8+ and Natural Killer (NK) cells were associated with better survival outcome [44, 45]. It is worth noting that stromal cells secreted growth factors responsible for the activation of Wnt signaling in the nucleus. Wnt ligands secreted by tumor cells would drive the phenotype of tumor associated macrophage towards M2 subtype which is considered anti-inflammatory via the Wnt signaling pathway, resulting in the tumor progression [46]. Consistent with the above descripted tumor-stromal-immune cell cross-talk, we found cluster1 with worse prognosis enriched in M2 subtype which is considered anti-inflammatory, furthermore, the expression level of M2 subtype was positively correlated with the risk score. Previous study reported tryptase-positive mast cells contributed in angiogenesis in the primary tumor of GC [47], however, our study found that resting mast cell was associated with worse prognosis. This opposite trend reflected that the ability of producing cytotoxic cytokines leading to the tumor degradation might also be stimulated [48]. To further unveil the potential role of m6A-related lncRNAs in guiding ICI therapy, we investigated the relationship of various biomarkers including TMB, MSI and IPS with our constructed risk model. TMB has evolved as an effective biomarker for recruiting patients who possibly respond to ICI therapy and patients with high TMB could gain better survival outcome from immunotherapy. MSI is a molecular indicator of defective DNA mismatch repair (MMR). MSI-H was correlated with favorable survival outcomes compared with MSS and MSI-L in GC [49]. MSI was demonstrated to be a robust indicator for immune checkpoint blockade therapy in the KEYNOTE studies [50, 51]. In our study, patients with low risk had higher TMB and accounted for larger proportion of MSI-H subtypes, which is consistent with the research conducted by Chalmers et al. [52]. Thus, the result suggested that patients in low-risk group more likely benefited in ICI therapy.

However, there are several limitations in our study. First, the efficacy of our model needs to be further validated in the external cohort with larger patient numbers. Second, since these selected m6A-related lncRNAs have never been reported in GC, the functions of these selected m6A-related lncRNAs should be confirmed further with functional experiments. Finally, the specific regulatory network between m6A and lncRNA, and their mutual roles of shaping TIME should be further unveiled.

## Conclusion

We conducted an in-depth bioinformatic analysis of the regulatory mechanisms of m6A-related lncRNAs in GC. M6A-related lncRNAs were screened out when integrating prognosis information. Two subtypes of GC patients (cluster1 and cluster2) based on different lncRNAs expression pattern were distinct in TIME characteristics, gene variants and OS. Moreover, prognostic m6A-related lncRNAs based risk score were highly associated with two subtypes, clinicopathological features and immune infiltrating cells. In addition, patients from cluster2 or low risk group were more likely to benefit from ICI therapy. The prognostic value of the risk model was validated in the external clinical cohort. These results provided new insights in GC evolutionary and development of immunotherapy.

## Supplementary Information

**Additional file 1.**

## Data Availability

Publicly available database analyzed in this study can be found in the The Cancer Gernome Altas (https://portal.gdc.cancer.gov/). The dataset of external cohort of our hospital is available from the corresponding author upon reasonable request.
