# Supplementary material for: Comprehensive analysis of tumor immune microenvironment and prognosis of m6A-related lncRNAs in gastric cancer
Source: BMC Cancer. 2022 Mar 24;22:316. doi: 10.1186/s12885-022-09377-8 (PMC8943990; doi:10.1186/s12885-022-09377-8)
Supplement: Supplementary file 1 — Additional file 1. [file 12885_2022_9377_MOESM1_ESM.docx]

# Supplementary Figures and Tables

**Table S1. Sequences of qPCR primers to detect RNA expression**

| Gene name | Gene ID | Chr | Coordinate | Primer sequence (5'->3') | |
| --- | --- | --- | --- | --- | --- |
|  |  |  |  | Forward primer | Reverse primer |
| AL139147.1 | lnc-INSL5-2 | 1 | 66665864-66677027 | AGCCTCTACCAATGTGATGCG | TCGTCATTCCCGACTACGTT |
| AL590705.3 | lnc-CTSV-3 | 9 | 97195338-97200041 | TGTGGTACATCAGGGAACTCAC | CTTTCCTTGGCAGAACCACAATT |
| AC022031.2 | lnc-RAB27B-11 | 18 | 55721063-55759122 | TCCCTACAAGACTGAAGCACCA | CCTCGTGTTCAGGACAAGCA |
| LINC00106 | LINC00106 | X | 1392421-1397006 | AGACTTCAGGCTTCATCGGAC | CTGCTCCATCTCAGGTGACCA |
| AL355574.1 | lnc-KCNT1-1 | 9 | 135907812-135913513 | AGCCGTCTGAAAAGAACACGA | TGTAGAATAAGCCAATGTCCACT |
| AL512506.1 | Lnc-ENOX1-4 | 13 | 43877715-43878163 | ACAATGGTGGGATTCGTATTCT | ACAAACTCTCTTCCCTGACAAC |
| AC005586.1 | lnc-RARRES2-2 | 7 | 150363777-150372590 | GATTCCTGAGCCTTTATGCCAG | CTTGTCAAAAGCTGAGACCGT |
| AP000873.4 | lnc-ANKRD42-1 | 11 | 83180144-83184520 | AGAGATAATGGGTTGGAGGCA | AACCATCACCCTCAACACTAG |
| GAPDH |  | 12 | 6534517-6538371 | ACAACTTTGGTATCGTGGAAGG | GCCATCACGCCACAGTTTC |
| TIM-3 | HAVCR2 | 5 | 157085832-157109044 | CTGCTGCTACTACTTACAAGGTC | GCAGGGCAGATAGGCATTCT |
| PD-L1 |  | 9 | 5450542-5470554 | TGGCATTTGCTGAACGCATTT | TGCAGCCAGGTCTAATTGTTTT |
| CD69 |  | 12 | 9752486-9760901 | ATTGTCCAGGCCAATACACATT | CCTCTCTACCTGCGTATCGTTTT |
| CTLA4 |  | 2 | 203867771-203873965 | GCCCTGCACTCTCCTGTTTTT | GGTTGCCGCACAGACTTCA |


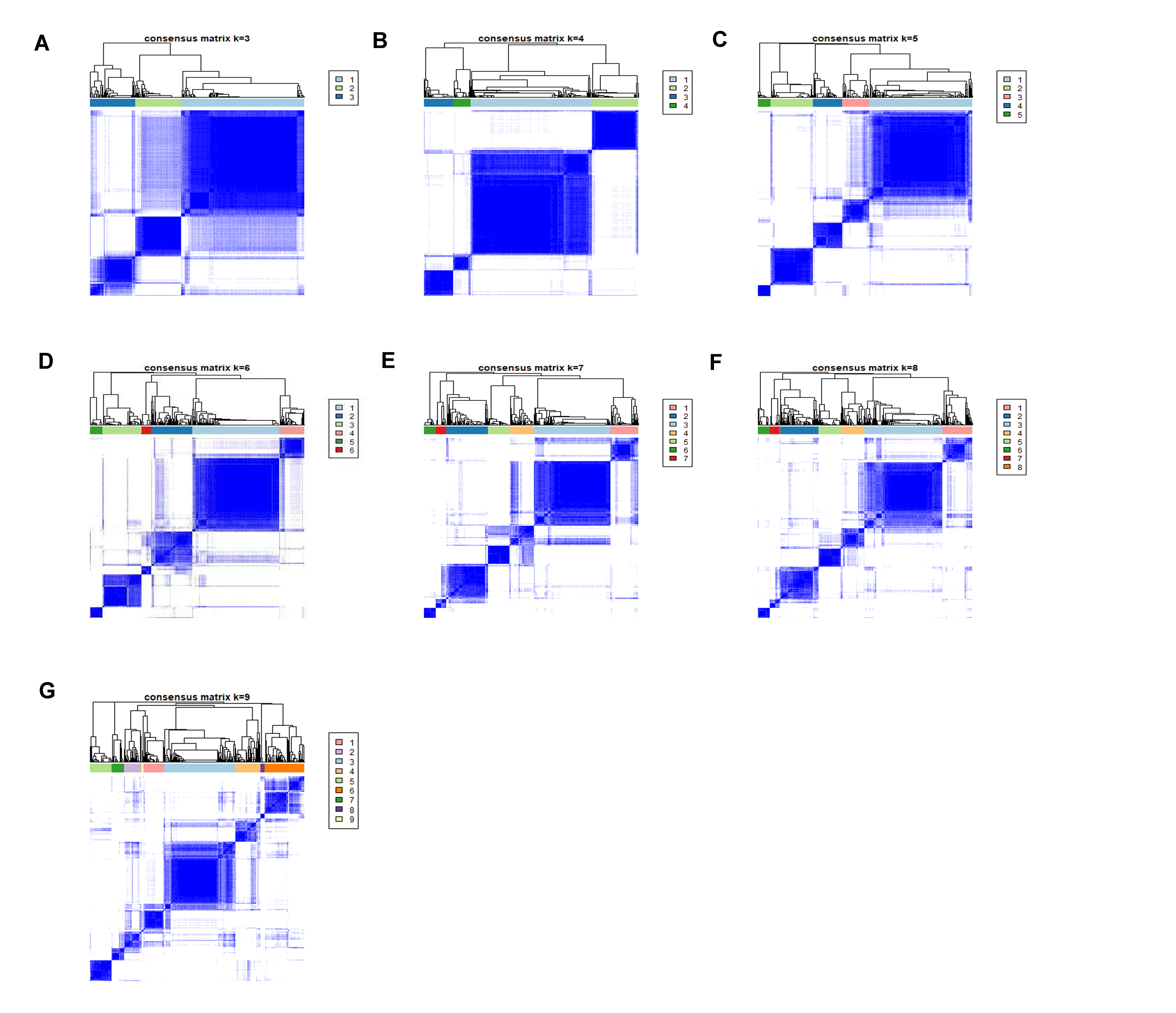


**Fig. S1.** Consensus clustering of GC samples based on m6A-related lncRNAs. (A-G) Consensus score matrix of all samples when k=3 to 9.


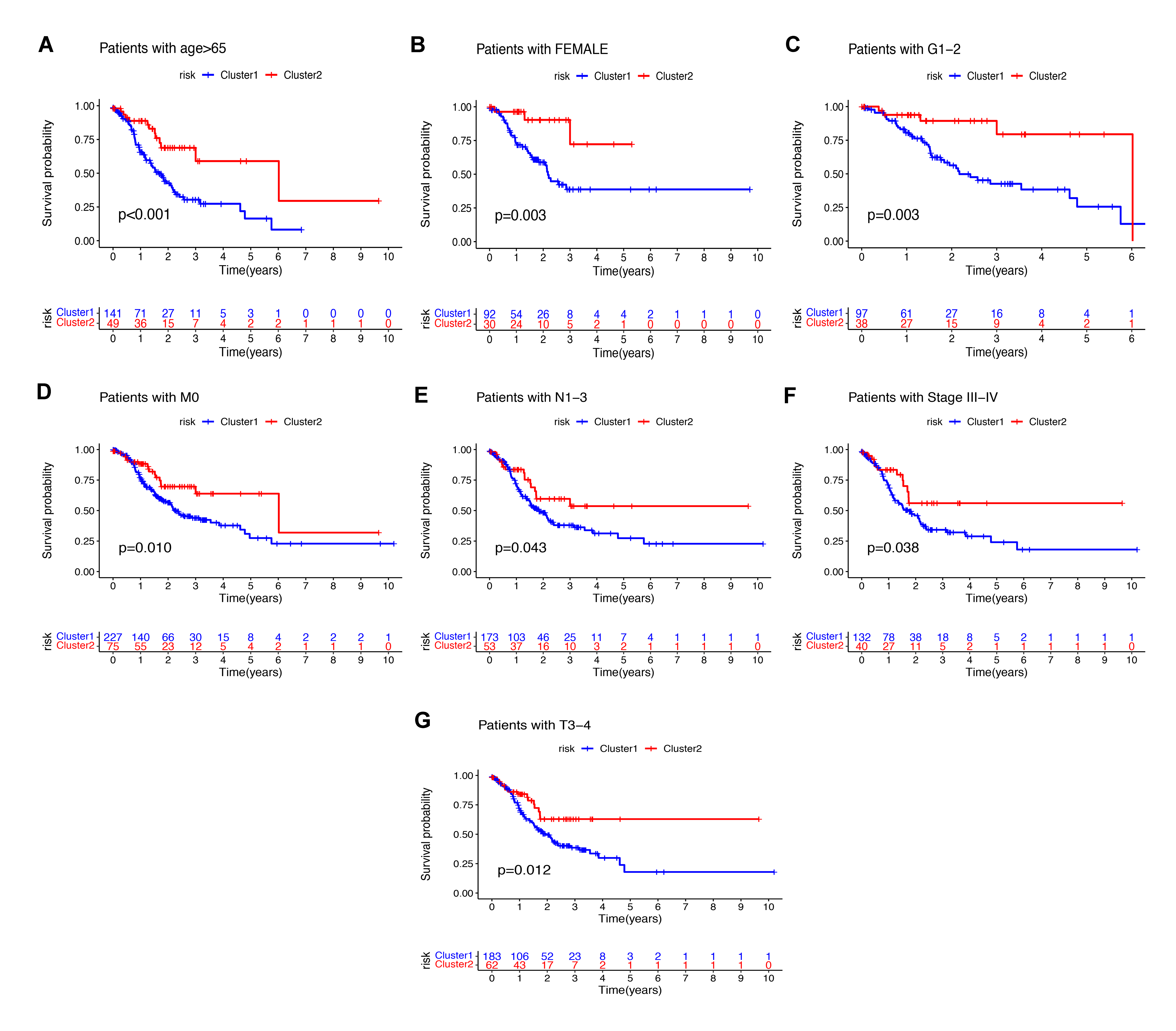


**Figure S2.** Subgroup Kaplan-Meier analysis in cluster1/2 group. (A-G) Survival analysis of cluster1/2 group in the samples of age more than 65 (A), female (B), G1-2 (C), M0 (D), N1-3 (E), stage III-IV (F) and T3-4 (G).


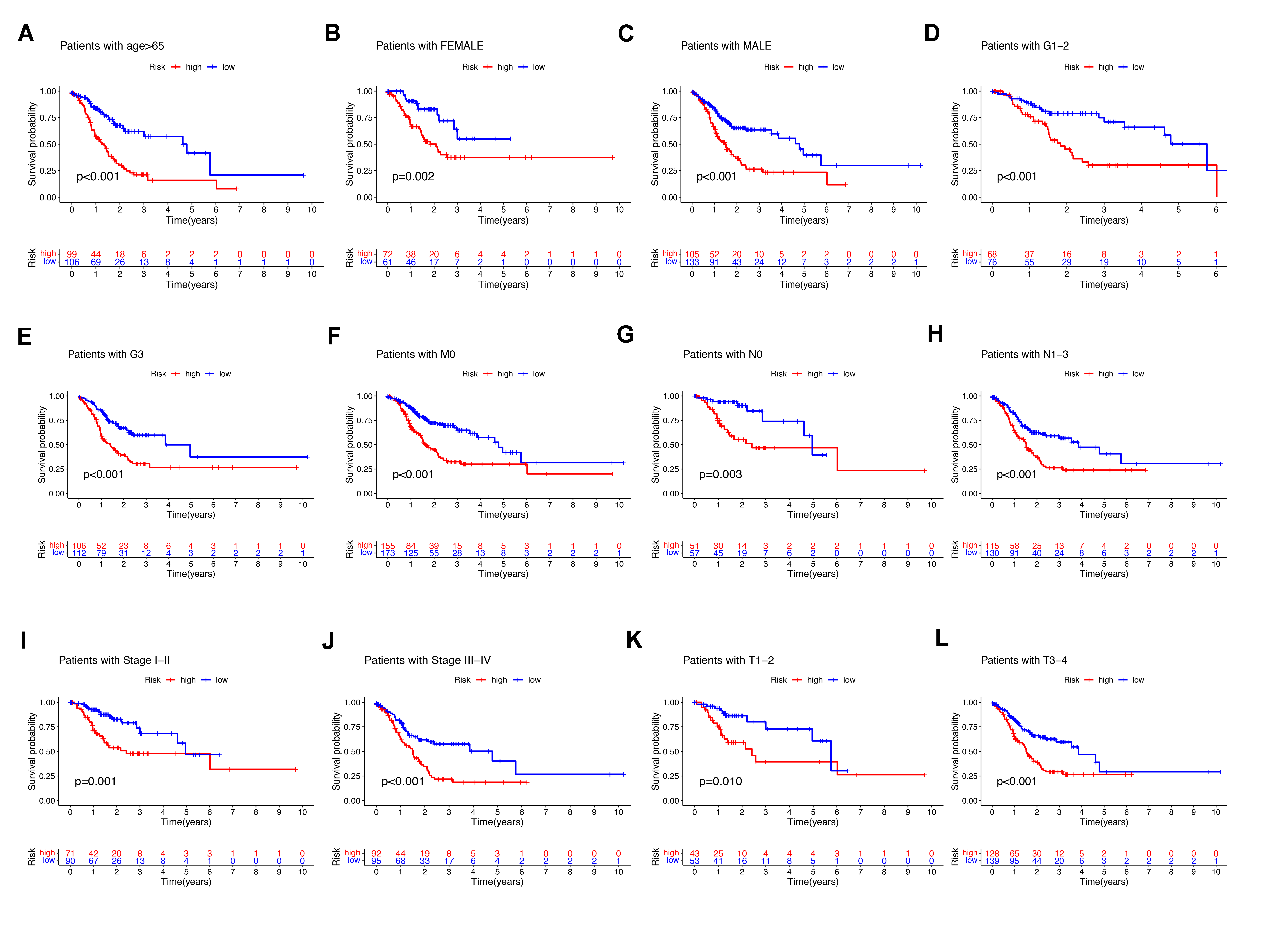


**Fig. S3.** Subgroup Kaplan-Meier analysis in different risk groups. (A-G) Survival analysis of low/high risk group in the samples of age more than 65 (A), female (B), male (C), G1-2 (D), G3(E), M0 (F), N0 (G), N1-3 (H), stage I-II (I), stage III-IV (J), T1-2 (K) and T3-4 (L).

**
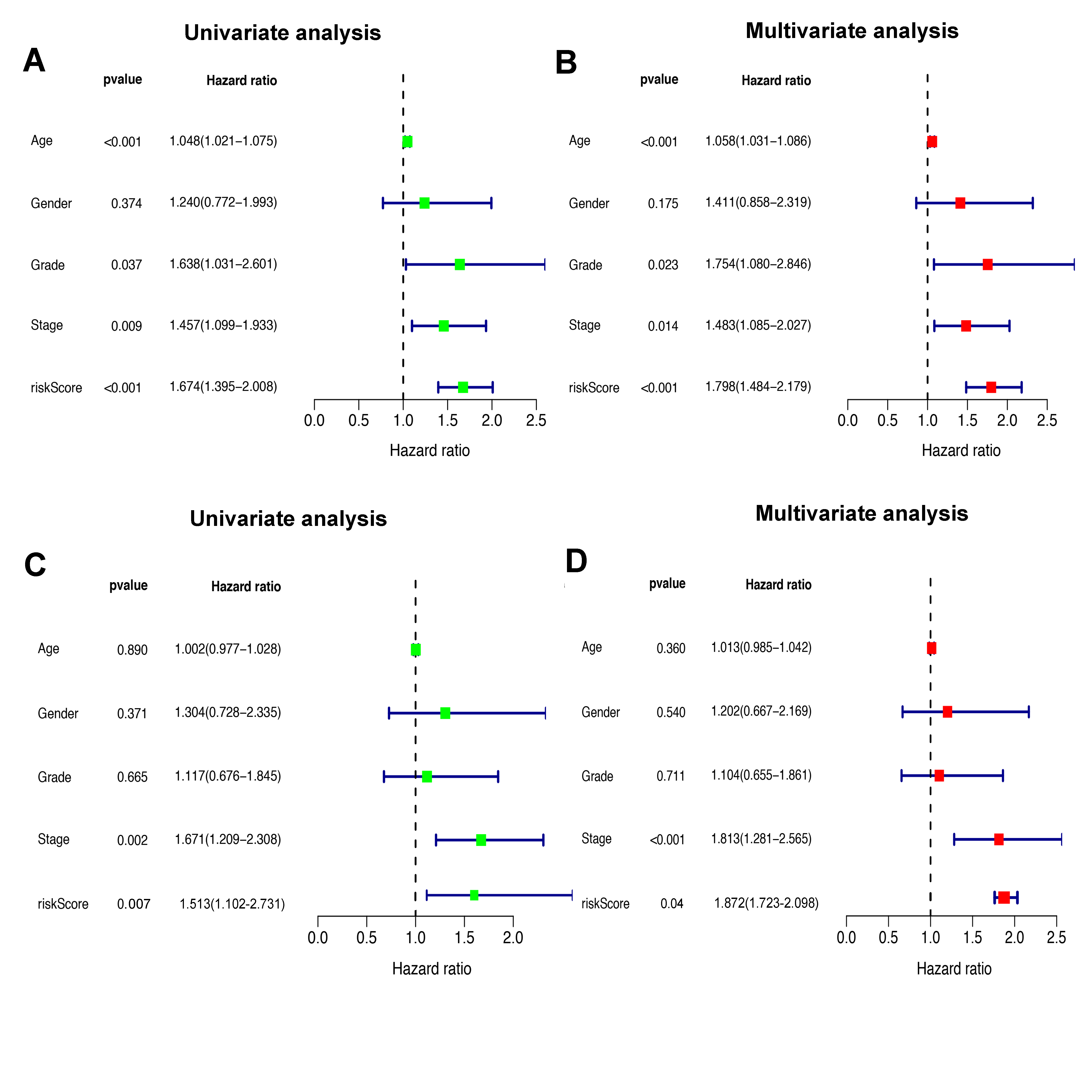
**

**Fig. S4.** Risk score based on m6A-related lncRNAs was an independent prognostic indicator. (A-B) Univariate analysis and multivariate analyses of risk score and several clinical variables associated with the prognosis of GC in the training cohort and (C-D) validation cohort.
